# Impact of Individual and Job Characteristics on Nurses' Scope of Practice in Spanish Hospital Units

**DOI:** 10.1155/2024/4796716

**Published:** 2024-03-18

**Authors:** Amaia Saralegui-Gainza, Paula Escalada-Hernandez, Cristina García-Vivar, Leticia San Martín-Rodríguez, Nelia Soto-Ruiz

**Affiliations:** ^1^Department of Health Sciences, Public University of Navarre (UPNA), Avda. Barañain s/n, Navarra, Pamplona 31008, Spain; ^2^University Hospital of Navarra, C/Irunlarrea 3, Navarra, Pamplona 31008, Spain; ^3^IdiSNA, Navarra Institute for Health Research, Irunlarrea, 3, Navarra, Pamplona 31008, Spain

## Abstract

**Background:**

Nurses are one of the largest and costliest groups in healthcare organizations; therefore, it is important to comprehend their scope of practice.

**Aim:**

To contribute to the improvement of nursing resource management in medical-surgical and Intensive Care Units by identifying factors that influence the scope of nursing practice. The hypothesis was that the activities carried out by nurses in medical-surgical units and intensive care units are influenced by individual and job-related factors, with job characteristics having an additive and moderating effect on individual characteristics.

**Materials and Methods:**

Cross-sectional correlational design to test the relationship between the individual and job characteristics on the nursing scope of practice measured by the Actual Scope of Nursing Practice (ASCOP) questionnaire. The sample consisted of 270 nurses. Linear mixed effects models analysis (LME) was used with the aleatory effect of the intensive care unit (ICU).

**Results:**

Belonging to the ICU decreased the scope of practice of nurses. We found a statistically significant effect of psychological demand, practice environment, role ambiguity, and growth need strength on the scope of nursing practice. The models explained a variance up to 24%.

**Conclusions:**

Although the survey results revealed the existence of broader scope of practice levels in Spanish hospital units than in the original Canadian study, the use of scope of nursing practice remains suboptimal. Higher levels on the psychological demand, the practice environment and in the individual growth need strength were related with a broader scope of practice. Otherwise, role ambiguity negatively affected the scope of practice. *Implications for Nursing Management*. This article provides an analysis of the impact of individual and job-related characteristics on the nursing scope of practice. It serves as a valuable resource for both managers and nurses, offering insights to improve nurses' working conditions and obtain more efficient workforces.

## 1. Introduction

In healthcare organizations, nursing human resources represent one of the most important groups because they are one of the largest and most expensive staff for healthcare services [1]. In addition, due to the current world situation, healthcare systems are facing a nursing staff shortage situation. There are insufficient numbers of nurses and that can directly impact patient outcomes [2]. McGahan et al. [3] have linked nursing human resources with a considerable improvement in patient outcomes, while it has been shown that a higher nursing ratio or lower staffing levels may compromise patient safety [4] and their association with adverse events as well as mortality rates in medical and surgical inpatient care [5].

Numerous studies provide evidence of the significant impact of effective nursing management on healthcare organization outcomes and address the sustainability of nursing services, that it is “strongly influenced by the availability of productive nurses” [6]. Even though the lack of nurses is a serious problem in healthcare administration, it can be an ideal period to analyze the efficiency of nursing human resources and the use of their full range of skills. Based on this analysis improvements in their work conditions may be proposed, and measures to ensure that they work as effectively as possible, and they provide continued and high-quality services can be adopted.

However, beyond the number of nurses working in each unit, it is important to consider the specific functions performed by these nurses. In this regard, a study developed by Gravling and Phoenix [7] confirmed that nurses stop performing nursing care while they carry out other tasks that are not within their competence. Therefore, it is imperative not only to examine coverage levels of nurses and their education qualifications in relation to the functions they are educated for and competent to carry out but also to analyze their specific functions and responsibilities, what is called “scope of nursing practice.”

The “actual scope of practice” refers to the professional activities carried out in a setting by nurses, as opposed to those that are expected to be optimal [8]. According to White et al. [9], the optimal scope of nursing practice, which is associated with the nurse's education level, job title, and experience, differs from the actual scope of nursing practice, which is influenced primarily by organizational context and employer policies.

In the context of a study carried out in the United States, the recommended strategies to reduce the cost generated by nurses, same as those focused in the improvement of quality of care, should be designed to ensure that nurses allocate the required time to patients and reduce activities that do not add additional value [10]. Non-value-added care is the care that is either unimportant, potentially harmful, or could be performed at a similar cost by less-trained or less costly staff.

Furthermore, the study developed by Déry's et al. [8] concluded that different levels in the scope of practice can influence not only healthcare costs but also patient outcomes and the satisfaction of nurses. These authors developed the Actual Scope of Nursing Practice (ASCOP) model, which explains how individual and job characteristics have a direct impact on the functions performed by nurses and on their job satisfaction [11].

The model was developed defining linkages between factors influencing the scope of nursing practice, where the personal characteristics such as growth need strength, education level, experience level and autonomy, psychological demand, and role stressors as job characteristics [8]. The model has been further evolved and different updates have been published defining the different variables. The same researchers independently studied the influence of the level of education and they concluded that nurses with higher levels of education were found to be those who perform the more complex nursing activities, both those involving higher management skills and those involving more committed tasks such as staff supervision [12]. In addition to educational level, growth need strength was found to be significant in predicting scope of nursing practice, while job autonomy, psychological demands, and role stressors were also associated as predictor variables [13].

The authors' latest update was conducted at a mother-infant hospital in Quebec. It included the incorporation of missed care and an analysis of organizational indicators [14]. Our proposal was to validate the same model in the Spanish population, so we considered including the variables used in the original framework. We did not include analysis of missed care, but we added some organizational factors, which we explain below.

Building upon the ASCOP model, the present study examined two new variables: nurses' evaluations of their practice environment and nurse-to-patient ratios. The practice environment consists of the organizational features of work that may facilitate or hinder nursing practice. This includes factors such as nurse-physician relationships, leadership and support for nurses, nurse involvement in hospital affairs, nursing's basis for quality care, and perceptions of resource provision and adequacy of resources [15]. On the other hand, nurses per patient ratio are a crude indicator of the nursing time available on a unit and refers to the number of patients nurses can care for simultaneously. The rationale for including these factors is based on their repercussion on both patients safety outcomes and the work factors of the nurses themselves. Numerous studies show that nurse staffing and the quality of nurses' work environment are associated with the quality of patient care. Hospitals with poor work environments present an increase of adverse outcomes but also lower levels of nurses' productivity [16]. Therefore, this variable, which not only impacts on patient outcomes, is particularly important in our study because of its direct influence on nurses' productivity, consequently affecting their scope of practice and their intention to leave their job [17].

**Figure 1:**
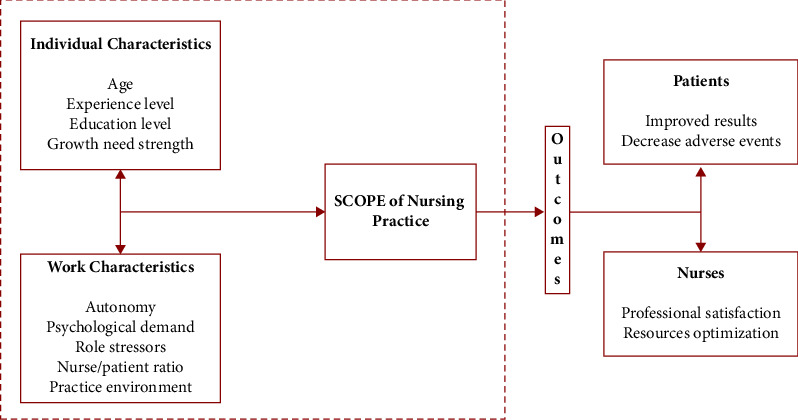
Redrawn conceptual framework based on ASCOP model [8].

When examining the factors that determine the scope of practice, the nurse-to-patient ratio was considered essential because, as Clarke argued, staffing levels directly affect the amount and quality of work that nurses are able to offer each patient [18]. Moreover, the same paper highlighted the relevance of studying the possible correlation between hospital work environment and staffing. Along the same line, Aiken et al. [19] have already worked on the implications of nurse staffing for recruitment, retention of nurses, and quality of care, suggesting that those job positions with better work environments were associated with a decrease in burnout and job dissatisfaction. This made the study of the working environment indispensable for our analysis. The framework for the current study is shown inside the die-cut box of Figure 1.

As shown in the area marked with the pointed box in Figure 1, our study focuses on how personal and work characteristics affect the scope of nursing practice, in addition to the effect that individual characteristics can have on work characteristics. In recent years, nursing managers are facing increasing difficulties in expanding nursing staff. This is due to a variety of factors, such as financial shortages, the underutilization of nurses, and the increasing cost generated by the new health needs of the population. Based on the above conceptual framework, our study seeks to find an approach that can guide nursing leaders to apply the necessary adjustments in nursing teams and to consider how to facilitate their working conditions to improve their productivity.

Regarding the hospitalization units, where the study took place, it is worth mentioning the particularities of the ICUs for nurses working in these settings. Working in such a specialized environment like the ICU can be particularly stressful for the staff [20], and several studies have demonstrated a connection between ICU nurses and increased level of burnout [21].

Furthermore, it should be highlighted that the nature of nursing workforce in Spain differs from the characteristics of the professional structure in other countries. In the Spanish context, the access to jobs in the different health services or fields is the same for all registered nurses regardless of their level of training, except in special cases such as mental health nurses and midwives, which are not included in the study. The nurses working in the hospital hold a university degree to access the nurse jobs and maintain the same level of qualification throughout their careers, even as they pursue training through postgraduate, master´s, and doctoral programs.

Considering the specific characteristics of nursing job in Spain, as well as the knowledge gap in this field, the main aim of this study is to contribute to the improvement of nursing resource management in medical-surgical and intensive care units by identifying factors that influence the scope of nursing practice.

Given the reasons described above and based on the following hypotheses, “the activities carried out by nurses in medical-surgical units and ICU are influenced by individual and job-related factors, with job characteristics having an additive and moderating effect on individual characteristics,” these research questions motivated the study: Q1: “How do nurses' individual characteristics and the nature of their work affect their professional activities?” Q2: “Are the individual and job characteristics determinants of the scope nursing scope of practice?” Q3: “What is the relationship between the individual characteristics of nurses and the characteristics of their work? And how do these factors affect the activities they engage in within their profession?”

## 2. Materials and Methods

### 2.1. Design

A cross-sectional design [22] was used to test the relationship between the individual and job characteristics of the nurses and the outcome variable, nursing scope of practice.

### 2.2. Setting

This study was performed in 29 adult hospitalization units (24 general medical and surgical units and 5 intensive care units) of the 3 main public hospitals in a Spanish region of “Region blinded for review.”

### 2.3. Sample

The following parameters were used to calculate the sample size: type I error of 0.05, type II error of 0.2 (power of 80%), and minimum detectable effect for a moderate intensity relation between the variables of interest 0.35. In addition, a 5% nonresponse rate was considered. The resulting size was penalized to contemplate the rest of the variables of the study, as suggested by Hsieh et al. [23], estimating a coefficient of determination for all of them of 0.32, according to the original study results [24]. Considering all these factors, a simple sample size of 270 subjects was calculated.

Convenience sampling was used. The total of 402 nurses working in clinical roles or in direct patient care were invited to participate in the study, and workers with less than 6 months of service at the institution were excluded. The first 270 nurses who responded to the questionnaire were chosen from among all eligible nurses.

### 2.4. Variables and Instruments

An online questionnaire was developed including the different measurement instruments to assess the study's variables of interest (Table 1).

The nursing scope of practice, or main variable, refers to the set of functions and responsibilities carried out by nurses, in relation to their competence and experience. It was measured with the ASCOP questionnaire [24], in its Spanish version [25]. The original questionnaire evaluates the frequency of activities carried out by nurses in six areas of practice: assessment and care planning, teaching of patients and families, communication and care coordination, integration and supervision of staff, quality of care, and patient safety and knowledge updating and utilization. The internal consistency of the original Canadian instrument showed Cronbach's alpha of 0.89 [24]. In this study, the Spanish adaptation of the ASCOP questionnaire was used, which includes 20 items and two main dimensions (Cronbach's alpha of 0.90 for the global score of the questionnaire and Cronbach's *α* of 0.875 for the first factor and 0.825 for the second) and was cross-culturally adapted following the Canadian version [25]. Regarding the definition of the characteristics of the scale, each item can be scored from 1 to 6, depending on the level of implementation of the scope of practice in each assumption, in relation to the frequency that nurses describe carrying out the activities of their responsibilities. In the case of answering 1, it would refer to this activity being performed “never,” while in the case of performing it “always,” it would be indicated with 6. The final score of the questionnaire results from the average of each of the questions. Therefore, a score of 3 or less would suggest that the development of the scope of practice is suboptimal. In the best scenario, the maximum score would be 6, and scores above 3 would indicate that nursing activities are being performed more frequently, so the scope of practice is being fully implemented. The two dimensions that constitute the cross-cultural adapted version in Spanish were used, as it was designed for this cultural context of this study.

The independent variables included individual and job characteristics.

Individual characteristics:

- Age (in years).
- Experience level in the current department (in years).
- Education level (graduate or postgraduate).
- Growth need strength: It is a personal characteristic that explains the variability between employees with respect to the need for self-actualization, learning and personal development at work [31]. It was measured with the validated Spanish version of the Job Diagnostic Survey (JDS) using two of its dimensions [26]. Cronbach's alpha obtained acceptable values: 0.91 for the first dimension and 0.72 for the second. It is divided into two dimensions in which both offer different response options in which the individual chooses the characteristics he/she would value when choosing a job. The first dimension evaluates job characteristics that the worker appreciates or would like to have in his job. The second subscale offers two response options to the individual that represent two specific conditions of his job; in it, he has to answer which of the two he would prefer to choose. Both measure those personal characteristics that allow assessing the need for self-development at work. A higher score on the scales means that the need for development at work is considered to be high.

Work characteristics.

- Autonomy and Psychological Demand: It refers to the ability to choose how to do the job and participate in decisions, and the amount of intellectual demands at work. It was measured using two factors of the Job Content Questionnaire [32], translated and validated in Spanish by Escribà-Agüir et al. [27], one factor to measure the concept of psychological demand at work and another for the concept of autonomy. Cronbach's alpha was rated between 0.74 and 0.88, presenting good reliability.
- Role Stressors: Role ambiguity, role conflict, and role overload. Role ambiguity and role conflict are defined as the lack of clarity regarding responsibilities and the excess of demands on the worker [33]. On the other side, role overload is defined as a situation in which the demands on the worker are excessive [34]. Role ambiguity and role conflict were measured with the Spanish version Role Ambiguity Scale of Rizzo et al. [35]. The analysis supported the existence of a bi-factorial structure (role ambiguity factor and role conflict factor) that explained 56% of the variance and Cronbach's alpha displayed acceptable value of 0.91. The Spanish version of the National Institute of Occupational Safety and Hygiene [28] was used. For the role overload, we used the Role Overload Questionnaire, developed by Beehr et al. [36] using the Spanish adaptation of Acosta [29]. The internal consistency reliability for role overload was alpha >0.95 for all facets, demonstrating a high correlation of the items.
- Practice environment: It is defined by Lake et al. [15] as factors that allow nurses to practice to the full scope of clinical practice and deliver safe, quality care to patients. The Practice Environment Scale-Nursing Work Index (PES-NWI), which has been adapted to Spanish by Orts-Cortés et al. [30], was used. The questionnaire showed acceptable internal consistency with Cronbach's alpha between 0.71 and 0.84. The tool has been recommended for its good psychometric properties and ability to compare results between studies and across different countries [37]. It was used in one of the most significant studies in nursing management research, RN4CAST [38], and is also included as a screening indicator for hospital staffing effectiveness in the Joint Commission accreditation standards [39].
- Nurse-to-patient ratio: Described as the minimum number of nurses in charge of a specified number of patients. The variable was assessed with a single item asking about the number of patients assigned to them on the last shift [40].
- Unit characteristics: Medical, medical-surgical, surgical, and belonging to ICU or not.

### 2.5. Data Collection

An online questionnaire was developed and conducted through the web platform SurveyMonkey©. Participants were recruited via e-mail with the help of nursing managers of each unit, who were responsible for sending the invitation to participate through the corporation mail address in order to preserve their privacy.

The message included information about the project, the consent for participating and the link to the online questionnaire. For maximizing the response rate, two personalized reminder e-mails were sent and the participation was incentivized with a lanyard as a gift, distributed anonymously after questionnaires returned by each unit nurses' managers [41].

The final survey, with 120 items, included 7 measuring instruments: five to measure job characteristics, one to measure individual characteristics, the demographic data questionnaire and the ASCOP questionnaire to measure the principal variable.

### 2.6. Data Analysis

Data synthesis and analyses were performed using R version 4.2.2. software. The sample was described using numbers and frequency for categorical variables and mean, standard deviation, minimum, and maximum for continuous variables. Before starting the multilevel analysis, the assumptions of normality of the distribution were examined. The assumptions on which multiple linear and logistic regression analyses are based were checked.

This study focuses on estimating the nursing scope of practice related to nurse's individual and work characteristics. LME [42] was used to determine the significance of the nurse's characteristics in relation to their scope of practice and to estimate the additional effect of the unit, ICU or not ICU. LME, or also called multilevel models or hierarchical models, are extensions of linear regression models that include random effects and correlated errors.

This statistical approach of hierarchical models with random effects assumes that subjects within the same unit type have internal consistency and a hierarchical structure, where individual nurses are nested within hospitalization units or ICU. In our model, the individual and work characteristics were included as fixed effects and a random intercept per nurse was included in the models to adjust for clustering of measurements within belonging or not to ICU.

The percentage of variance in care time explained by the mixed models (R2) was estimated using the method described by LaHuis et al. [43]. We used R software version 4.3.1. [44] with the lme4 [45] package to perform linear mixed analysis. The level of significance was set as 0.05.

### 2.7. Ethical Considerations

One-time anonymous survey was performed. The objectives of the study were explained and participants gave free consent to their participation. The study obtained the approval from the Ethics Committee of the (blinded for review) Public University of Navarre (PI: 005/19).

## 3. Results

We obtained 310 questionnaires and selected the first 270 questionnaires completed entirely to the end by nurses working on hospitalization and ICU. The data imputation process was dismissed considering the potential impact on the analysis due to the substantial amount of missing data in each incomplete questionnaire (more than 90% of the items) and for avoiding significant biases. Furthermore, the literature suggests that the benefits of using imputation in cases of mixed-model analysis are not necessary [46]. The response rate was 67.16%, and although there is no consensus on the appropriate response rate [47] (Morton, 2012), mixed-model designs with higher response rates benefit more than larger sample sizes [48]. However, a recent meta-analysis found that a response rate of 67% is recommended for health sciences studies [49].

### 3.1. Description of the Variables

Following the conceptual framework of the study, we present the description of the variables. Concerning to the participants' individual characteristics, the nurses had an average age of 39.9 years (SD = 9.3), and the mean of years of nursing experience was 11.6 years (SD = 8.6). 18.75% (*n* = 58) of our sample had completed the basic nursing degree with any postgraduate degree, but 81.3% (*n* = 252) had only the nursing degree. About the last individual independent variable, measured by the growth need strength questionnaire, the mean of “would like” variable was 8.77 (SD 1.01) and the mean of “job choice” section 2.58 (SD 0.36). About the characteristics of each unit, 34.8% (*n* = 94) of the nurses worked on an intensive care unit, while 65.2% (*n* = 176) were assigned to hospital units. Otherwise, 23.3% (*n* = 63) worked at a medical unit, 18.9% (*n* = 51) at a surgical unit, and 57.8% (*n* = 156) in mixed medical-surgical unit. A total of 19 different hospitalization units constituted our sample. The first work characteristic, autonomy was 2.80 (SD 0.30), psychological demand obtained a mean of 3.05 (0.290), role conflict and ambiguity averages were 2.76 (SD 0.93) and 3.50 (SD 1.28), and the mean for role overload was 3.15 (SD 1.18). The mean of nurse-to-patient ratio was 6.72 (SD = 3.9), and finally, the mean for the practice environment was 2.76 (SD 0.39). Higher scores were related to higher levels of each variable. Regarding the total mean scores of our principal variable, the scope of nursing practice obtained a mean average of 4.05 (SD 0.72) (Table 2).

### 3.2. Linear Mixed Effects Models

**Figure 2:**
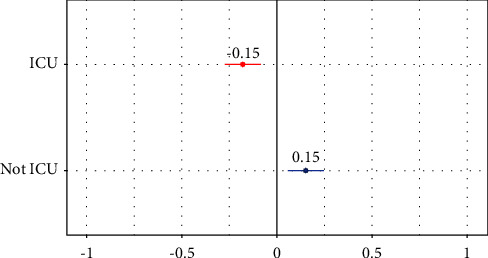
Estimated aleatory effects by ICU.

Random ICU effect was included. Belonging to the ICU decreased the scope of practice of nurses in 0.15. Aleatory effects of analyzed unit are shown in Figure 2.

The results of the LME analyses conducted with the confounding variables and with adjustment for the corresponding values. As shown in Table 3, the estimated random effects for nursing scope of practice were 0.605 for between-units variance.

**Figure 3:**
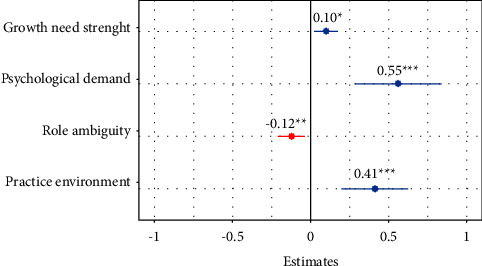
Fixed effects of LME. Significance levels are specified with ^∗^, ^∗∗^, and ^∗∗∗^.

We found a statistically significant effect of psychological demand (0.561, *p* < 0.001), practice environment (0.414, *p* < 0.001), role ambiguity (−0.121, *p*=0.005), and growth need strength (0.099, *p*=0.010) on scope of nursing practice. The models explained 24% of the variability within nurses' scope of practice (*R*_1_^2^ approx. 0.24). Fixed effects are presented in Figure 3.

All the variables were positively related with the nursing scope of practice except from role ambiguity, that demonstrated a negative trend, so that nurses who showed greater punctuation on those variables were related to a lower scope of practice (−0.12).

Otherwise, nurses with practice environment that multiplied the probability of having upper scope of practice by 0.41, and it occurred the same with the growth need strength that also predicted (0.10 times) the nursing scope of practice. Curiously, nurses with more role psychological demand had 0.56 times higher probability of developing their full scope of practice.

## 4. Discussion

This study examined the association of the individual and job characteristics on nursing scope of practice on hospital units. First, in this study, the scope of nursing practice (E-ASCOP 4.05) showed higher values than the original study (ASCOP 3.47) [24]. This difference may be explained, on the one hand, by the nature of unit in which the studies were carried out. The Canadian research was conducted in pediatric units, while in our case, we focused on hospital units. Country-specific differences in employment, the legislation and regulation of nursing practice and the level of training of nurses may be another key reason for these variations in scores. The result in our setting, scoring 4.05, shows that the frequency with which nurses performs tasks within their scope of practice is adequate but not optimal, still far from reaching the maximum ASCOP score of 6.

In the study of the nature of the scope of nursing practice, it is important to consider that while nurse work according to a framework of competencies set out in the ASCOP questionnaire, each hospital unit has its own particularities. Therefore, the activities carried out by nurses may differ between different inpatient units, including the ICU, as well as the working environment are conditioned not only by job-related variables but also by the work colleagues themselves. This is why we chose this methodological approach, which considers the effect that belonging to the same unit may have. We consider that nurses working in the same unit will have similar characteristics. In this line, our results show that belonging to ICU decreases nurses scope of practice in 0.15.

Following the model generated in this study, the variables of psychological demand (0.56), practice environment (0.41), role ambiguity (−0.12), and growth need strength (0.10) were the most important characteristics in predicting the scope of nursing practice. The first of these variables is psychological demand, and as in the original study, each increase in this variable has a positive effect on the scope of practice. According to other authors, psychological demands at work are one of the main stress factors perceived by workers, but it has also been demonstrated that this stress can be attenuated by an enhancement of decision-making and a correct definition of specific functions [50]. Contrary to what might be expected, nurses with higher psychological demand are the ones who implement their scope of practice the most. Nurses with higher psychological demand often possess a strong intrinsic motivation and drive to excel in their profession. They are more likely to seek out opportunities for learning, skill enhancement, and career advancement. This motivation pushes them to actively pursue professional development and expand their scope of practice [51].

A second variable determined as a strong predictor of scope of practice in our study is the practice environment, showing a negative effect on the activities carried out by nurses. There are also studies that associate a poor work environment with missed care and negative patient outcomes [15]. It is also known that most of those nursing activities that are omitted are related to comfort activities and communication with patients [52]. Similar results were found in a Turkish research, demonstrating how the practice environment is related to work performance levels and how the staffing has a direct influence on the nurses' scope of practice [53]. It is worth to mention that practice environment, one of the two new variables included in our framework, was found to be a predictor of the scope of nursing practice, as supported by studies such as Pearson's [54] which analyzed the relationship between healthy practice environments and better patient outcomes.

With the addition of the nurse's practice environment as a job characteristic, we intend to develop a predictive model describing the relationship between that variable and the nursing scope of practice. The introduction of that variable was intended to include the more contextual and environmental factors or aspects of the job characteristics, not considered before this study. Understanding the relationship between the practice environment and nurses' scope of practice is essential for optimizing patient outcomes, enhancing job satisfaction, and ensuring the delivery of safe and efficient care [55].

In addition, the obtained results suggest that role ambiguity has a negative relationship with nurses' scope of practice. Tarrant [56] explained that the principal cause of role ambiguity is the inadequate definition of the role, and it has several impacts on job performance. It could be outlined in the following explanation: as a nurse's role is broader, she/he is responsible for more extensive competencies and consequently presents greater performance of the scope of practice. Ambiguity in job roles can hinder nurses' ability to effectively perform their scope of practice. When nurses are uncertain about their authority, decision-making capacity, or the scope/boundaries of their role, they might be hesitant to assume certain responsibilities or may not fully utilize their knowledge and skills. This can limit the extent to which nurses can contribute to patient care and highlight a weakness of nurse's workforce management [57].

Finally, our findings confirm that, among the individual characteristics analyzed, the growth need strength or need of personal self-fulfillment is related to the scope of nursing practice, in contrast to what was published by Fuertes et al. [26]. The intensity of growth need strength reflects the motivation of workers to perform their jobs and their capacity to adapt to change. In our case, the “would like” subscale obtained higher scores (between 71 and 100%), while the “job choice” subscale scored low, indicating that attention should be paid to the nurses' work system. Nurses with high growth need strength seek opportunities for learning, skill enhancement, and career advancement. When nurses' growth needs are met, they are more likely to actively seek out and embrace opportunities to expand their scope of practice. Conversely, if growth needs are not addressed, nurses may become disengaged and less motivated to pursue professional development, leading to a narrower scope of practice [58].

Our model explained 24% of the variance of nurses' scope of practice, compared to 32.5% of the ASCOP model developed by Déry et al. [8]. The Spanish ASCOP model demonstrated the significant influence of four variables on the scope of nursing practice: one variable concerning the individual characteristics of the nurses (growth need strength) and three of the variables referring to job characteristics (role ambiguity, psychological demand, practice environment). In our case, work characteristics were the most sensitive predictors of nursing scope of practice, in contrast to an Australian research [59] which used the Scope-q questionnaire and demonstrated the influence of demographic characteristics rather than work characteristics.

As a main difference compared to the previous model and considering the statistical analysis carried out in the Spanish context, our results do not include the educational level, autonomy, and role overload as predictors of nurses' scope of practice, in contrast with Déry's original study [8], where these variables were three of the five variables included in the model. This could be due to cultural differences, differences in the working conditions of Spanish nurses, and therefore, in their scope of practice.

To optimize nurses' scope of practice, healthcare organizations and leaders must consider these factors. Creating a practice environment that minimizes psychological demand, reduces role ambiguity, and promotes growth need fulfilment is essential. Strategies may include providing adequate support systems, clear role expectations, opportunities for professional development, and fostering a culture of collaboration and shared decision-making. In addition, addressing workload issues, promoting work-life balance, and implementing policies that support nurses' well-being can contribute to a positive practice environment that enhances nurses' scope of practice.

Using mixed effects models within nursing intensive care units, researchers may gain insights into the complex interplay between individual nurse characteristics, unit-level factors, and outcomes. This knowledge can inform evidence-based practices, healthcare policies, and strategies for improving nursing care within healthcare settings. Future research should continue to explore these dynamics to further inform interventions and practices that enhance nurses' scope of practice and consider job-specific differences.

The random effect of UCI is justified for multiple reasons. First of all, such specific care units may show different behaviors and contrary to what might appear, the scope of practice performed by their nurses is lower than in inpatient units. This may be due to technological advances and increasing care needs in recent years, the specific competencies of ICU nurses have been increasing [60]. This acquisition of new responsibilities may have compromised the work beyond their scope of practice and focused ICU nurses on more job-specific tasks, such as the care of specific devices and complex equipment. Secondly, it is well known that nurses working in ICUs are subjected to higher levels of stress. There are even studies that state that these nurses experience a change in their temperament after started working in ICU [61]. These data could lead us to think that belonging to this type of unit has effects not only on the characteristics of the job itself but also on the more personal attributes of these workers.

### 4.1. Limitations of the Study

One of the main limitations of this study was the elevated number of items (120) that the nurses had to answer. Despite being an easy to understand questionnaire, the response time was approximately 20 minutes, so there were several participants who started the questionnaire but did not complete it.

On the other hand, in our context, no significant differences were found, probably due to the low variability among nurses in their educational level, since in Spain, most nurses hold similar educational degrees (a three or four-year university degree). A line for future research could focus on comparing the scope of practice of nursing managers and general nurses, in which these differences could be studied, because more specialized educational qualifications have begun to be required for these roles.

Moreover, this is the first research in Spain to measure the scope of practice and its determinants. For future projects, a multicenter study could be considered to compare the different regions of Spain, assessing how the policies of each healthcare regional service affect both nurses' working conditions and the scope of their practice. In addition, it would be valuable to explore the particularity of the ICU, potentially needing a distinct study approach.

## 5. Conclusions

In this study, we have determined that there are individual and job characteristics that predict the scope of nursing practice in hospital units. Mixed effects models allow researchers to better understand the relationships between individual nurses and hospital units, providing valuable insights for nursing practice, healthcare management, and policy decisions. This new statistical approach complements the original analysis by including the random effect of belonging to a particular unit (ICU or medical-surgical unit) on nurses' scope of practice, which has not been previously considered. Nurses belonging to same unit had similarities and the results are different between those groups.

The scope of practice of Spanish hospitalization nurses demonstrated a broader range of practice than that of Canadian nurses. In the model we present, the influence of psychological demand, practice environment, role ambiguity, and growth need strength were the predictive variables of the nursing scope of practice.

Although the results showed better levels of scope of nursing practice than in the original study, it remains suboptimal. With the identification of the factors that are determinants, we can propose strategies to improve them and thus facilitate nurses to implement more broadly their scope of practice.

## Figures and Tables

**Table 1:** Variables and the measurement instruments with their characteristics.

Variable	Questionnaire	N. items	Scale (Likert)
Nursing scope of practice	ASCOP questionnaire [25]	20	1–6, “never” to “always”

Age, years of experience, education level, nurse-to-patient ratio, unit	Demographic questionnaire		

Growth need strength “would like”	Job diagnostic survey (2 subscales) [26]	11	4–10
“Job choice”		12	1–5

Autonomy and psychological demand	Job content questionnaire [27]	18	1–4, “strongly disagree” to “strongly agree”

Role ambiguity and role conflict	Role ambiguity scale [28]	14	1–7, “strongly disagree” to “strongly agree”

Role overload	Role overload questionnaire [29]	3	1–5, “strongly disagree” to “strongly agree”

Practice environment	Pes-NWI [30]	32	1–4, “strongly disagree” to “strongly agree”

**Table 2:** Descriptive statistics.

		Scale	Mean	SD	Observed range	
					Min	Max
Scope of nursing practice	Global score	1–6	4.05	0.72	2.19	5.88
	First dimension	1–6	4.41	0.83	2.20	6.00
	Second dimension	1–6	3.60	0.79	1.70	5.80

Individual characteristics	Age (years)		39.9	9.3	23	59
	Experience level		11.6	8.6	1	35
	Education	81.3%	*n* = 252	—	18.7%	*n* = 58
	Graduate/postgraduate					
	Growth need strength					
	“Would like”	4–10	8.77	1.01	5.09	10.00
	“Job choice”	1–5	2.58	0.36	1.58	3.67

Work characteristics	Autonomy	1–4	2.80	0.30	1.67	2.67
	Psychological demand	1–4	3.05	0.290	2.33	3.78
	Role ambiguity	1–7	2.76	0.93	1.00	7.00
	Role conflict	1–7	3.50	1.28	1.00	7.00
	Role overload	1–5	3.15	1.18	1.00	5.00
	Nurse to patient ratio		6.72	3.9	1.00	20.0
	Practice environment	1–4	2.76	0.39	1.72	3.66
	ICU/hospitalization	34.8%	(*n* = 94)	—	65.2%	(*n* = 176)

**Table 3:** Linear mixed effects models analysis, including random unit effect.

Parameter	Estimate	95% confidence level		*p* value
		Lower bound	Upper bound	
Scope of practice				
Intercept	0.605	−0.540	1.749	0.300
Psychological demand	0.561	0.284	0.838	<0.001
Practice environment	0.414	0.202	0.625	<0.001
Role ambiguity	−0.121	−0.207	0.035	0.005
Growth need strength	0.099	0.023	0.1761	0.010

## Data Availability

The mixed effects models data used to support the findings of this study are available from the corresponding author upon request.

## Supplementary Materials

Supplementary Materials Completed STORBE checklist for observational studies [62].
